# *Soehngenia longivitae* sp. nov., a Fermenting Bacterium Isolated from a Petroleum Reservoir in Azerbaijan, and Emended Description of the Genus *Soehngenia*

**DOI:** 10.3390/microorganisms8121967

**Published:** 2020-12-11

**Authors:** Tamara N. Nazina, Salimat K. Bidzhieva, Denis S. Grouzdev, Diyana S. Sokolova, Tatyana P. Tourova, Sofiya N. Parshina, Alexander N. Avtukh, Andrey B. Poltaraus, Azhdar K. Talybly

**Affiliations:** 1 Winogradsky Institute of Microbiology, Research Center of Biotechnology, Russian Academy of Sciences, 117312 Moscow, Russia; salima.bidjieva@gmail.com (S.K.B.); sokolovadiyana@gmail.com (D.S.S.); tptour@rambler.ru (T.P.T.); sonjaparshina@mail.ru (S.N.P.); 2 SciBear LLC, 13617 Tallinn, Estonia; denisgrouzdev@gmail.com; 3 Skryabin Institute of Biochemistry and Physiology of Microorganisms, Pushchino Scientific Center for Biological Research of the Russian Academy of Sciences, 142290 Pushchino, Russia; avtukh@rambler.ru; 4 Engelhardt Institute of Molecular Biology, Russian Academy of Sciences, 119991 Moscow, Russia; abpolt@gmail.com; 5 Institute of Microbiology of the National Academy of Sciences of Azerbaijan, Baku AZ1073, Azerbaijan; ajdartalibli@mail.ru

**Keywords:** *Soehngenia longivitae*, new species, genome analysis, polyphasic taxonomy, oilfield

## Abstract

A methanogenic enrichment growing on a medium with methanol was obtained from a petroleum reservoir (Republic of Azerbaijan) and stored for 33 years without transfers to fresh medium. High-throughput sequencing of the V4 region of the 16S rRNA gene revealed members of the genera *Desulfovibrio*, *Soehngenia*, *Thermovirga*, *Petrimonas*, *Methanosarcina*, and *Methanomethylovorans*. A novel gram-positive, rod-shaped, anaerobic fermentative bacterium, strain 1933P^T^, was isolated from this enrichment and characterized. The strain grew at 13–55 °C (optimum 35 °C), with 0–3.0% (*w*/*v*) NaCl (optimum 0–2.0%) and in the pH range of 6.7–8.0 (optimum pH 7.0). The 16S rRNA gene sequence similarity, the average nucleotide identity (ANI) and in silico DNA–DNA hybridization (dDDH) values between strain 1933P^T^ and the type strain of the most closely related species *Soehngenia saccharolytica* DSM 12858^T^ were 98.5%, 70.5%, and 22.6%, respectively, and were below the threshold accepted for species demarcation. Genome-based phylogenomic analysis and physiological and biochemical characterization of the strain 1933P^T^ (VKM B-3382^T^ = KCTC 15984^T^) confirmed its affiliation to a novel species of the genus *Soehngenia*, for which the name *Soehngenia longivitae* sp. nov. is proposed. Genome analysis suggests that the new strain has potential in the degradation of proteinaceous components.

## 1. Introduction

The genus *Soehngenia* belongs to the family *Tissierellaceae* [1] of the order *Tissierellales* of the class (not validly published) *‘Tissierellia*’ [2] within the phylum *Firmicutes*. At the time of writing, the genus *Soehngenia* was represented by the type strain BOR-Y^T^ (=DSM 12858^T^ = ATCC BAA-502^T^) of the only published species *Soehngenia saccharolytica* isolated from an anaerobic sludge bed reactor treating potato starch waste [3]. Strain DSM 12858^T^ is a gram-positive, rod-shaped, motile, mesophilic, neutrophilic anaerobic spore-former, which ferments a wide range of carbohydrates and other carbon sources, including yeast extract, cysteine, and serine. Sulfite and thiosulfate may be used as electron acceptors (reduced to H_2_S). The strain performs dismutation of benzaldehyde to benzoate and benzyl alcohol [4]. Using 16S rRNA gene sequencing, members of the genus *Soehngenia* were detected in petroleum reservoirs [5,6,7,8] and in water droplets dispersed in heavy oil [9], in methanogenic crude oil- and propionate-degrading enrichments [10,11], in marine sediments contaminated with heavy metals and polycyclic aromatic hydrocarbons [12], and in cyanide-degrading enrichments [13].

In 1977, in the course of ecological investigation of microbial communities of the Binagady petroleum reservoir (Republic of Azerbaijan) [14], methanogenic enrichment cultures were obtained from production water samples. This enrichment was routinely maintained in our laboratory for 8 years by means of successive 3–6 month transfers into the medium with methanol. Fermentative bacteria and methanogens of the genus *Methanosarcina* were identified in the enrichments using culture-based techniques [15]. Since 1985, several flasks with the methanogenic enrichment growing on the medium with methanol have been stored at room temperature (18–24 °C) for 33 years without transfers to fresh medium in order to investigate survival of oilfield communities. In 2018, the enrichment was used to inoculate fresh medium with methanol, and methane production was registered in this culture. Sarcinae were present in the environment, as well as the cells of other morphological types. Methanosarcinae and other microorganisms occurring in the enrichment were isolated by inoculating the media with various carbohydrate and proteinaceous substrates, with or without antibiotics, and incubating the media at various temperatures. Thus, several stable enrichments were obtained from the methanogenic enrichment. One of the first isolates was a pure culture of a fermenting bacterium, strain 1933P^T^ (VKM B-3382^T^ = KCTC-15984^T^), straight motile rods growing in the medium with peptone at 28 °C [16]. The 16S rRNA gene sequence of the strain 1933P^T^ had 98.5% similarity to that of *Soehngenia saccharolytica* DSM 12858^T^. This value was below the threshold accepted for species demarcation at 98.65% 16S rRNA gene sequence similarity [17].

The present study aimed to describe the phylogenetic diversity of prokaryotes in methanogenic enrichment stored for 33 years and details of the isolation procedure, the morphological and chemotaxonomic properties of strain 1933P^T^, as well as the results of a comparative analysis of *Tissierellaceae* genomes. These data allowed us to assign strain 1933P^T^ to a novel species of the genus *Soehngenia*, for which the name *Soehngenia longivitae* sp. nov. is proposed.

## 2. Materials and Methods

### 2.1. Characteristics of the Sampling Site

The Binagady oilfield is located near Baku city in the Republic of Azerbaijan. Exploitation of this oilfield involves flooding with the Lake Beyuk Shor water (salinity 7–10 g L^−1^). In 1977, a water sample was obtained from production well 1933, which operated on a sandstone oil-bearing horizon, was located at a depth 590–597 m below sea level, and has a temperature of 28 °C. The total salinity of the studied formation water was 84.8 g L^−1^. The oil recovered was heavy, paraffin-free, with specific density of 0.899–0.964 g/cm^3^ (at 20 °C). Information on the physicochemical conditions and microbial numbers in production water of the Binagady oilfield was presented earlier [14]. The sample of oil–water mixture taken directly from production wellhead was collected in a sterile 0.5 L serum bottle. The bottle was sealed with a rubber stopper and screw cap and transported at ambient temperature to the field laboratory, where it was immediately used for inoculation of the nutrient media.

### 2.2. Isolation of Methanogenic Enrichment and Pure Culture of a Fermenting Bacterium

In 1977, a methanogenic enrichment culture was obtained in the mineral medium (MM) [18] containing, per liter distilled water, 0.2 g KH_2_PO_4_, 0.25 g NH_4_Cl, 15 g NaCl, 0.4 g MgCl_2_ · 6H_2_O, 0.5 g KCl, 0.1 g CaCl_2_ · 2H_2_O, 0.5 g Na_2_S · 9H_2_O, supplemented with methanol (5%, vol/vol), and inoculated with production water (10%, vol/vol) from the Binagady oilfield. Since 1985, the culture was stored at room temperature for 33 years without transfers to fresh medium. In 2018, using this long-term storage methanogenic enrichment as inoculum, a pure culture, strain 1933P^T^ (VKM B-3382^T^ = KCTC 15984^T^), was isolated by sequential transfers from the highest dilutions of enrichment to the mineral medium MM with peptone (2 g L^−1^), yeast extract (0.2 g L^−1^), NaCl (15 g L^−1^), and Na_2_S · 9H_2_O (0.2 g L^−1^), at pH 7.0 and 30 °C. The medium was supplemented with 1 mL L^−1^ of the following solutions: 0.1% (*w*/*W*) Mohr’s salt (FeSO_4_ · (NH_4_)_2_SO_4_ · 6H_2_O), vitamins [19], and microelements [20]. The medium was prepared anaerobically under a flow of Ar, dispensed into Hungate tubes [21], and sealed with butyl rubber stoppers. Strain 1933P^T^ was subsequently maintained in this medium. Strain 1933P^T^ did not form colonies in solid medium. The culture purity was confirmed by microscopic studies, as well as by the 16S rRNA gene and genome sequencing.

### 2.3. DNA Isolation, Amplification and Sequencing of the 16S rRNA Genes from the Methanogenic Enrichment

Cell biomass from 20 mL of the methanogenic enrichment culture stored for 33 years was collected on the membrane filter, washed off with a lysing solution containing 0.15 M NaCl and 0.1 M Na_2_-EDTA (pH 8.0) and used for DNA extraction. Isolation of the total DNA was carried out using the PowerSoil DNA Isolation Kit (MoBio, Carlsbad, CA, USA), according to the manufacturer’s recommendations. DNA was stored in a freezer at −20 °C. Total genomic DNA was amplified using the 515f/806r primer set that amplifies the V4 region of the 16S rRNA gene [22]. Sequencing was carried out on a MiSeq platform (Illumina, San Diego, CA, USA) using the MiSeq Reagent Kit v3 (600 cycles) (Illumina, United States) according to the manufacturer’s recommendations. The obtained 250-bp paired-end reads were further processed according to the workflow implementing suitable scripts from USEARCH version 10 [23]. Reads were demultiplexed (-fastx_demux), trimmed to remove the primer sequences (-fastx_truncate), and then quality filtered (-fastq_filter). UNOISE3 [24] was used to generate zero radius operational taxonomic units (zOTUs). zOTU is a term specific to analysis with UNOISE, referring to operational taxonomic units which were generated by an error correction algorithm as opposed to a sequence similarity clustering algorithm [25]. Raw merged read pairs were mapped back to zOTUs using the -otutab command. zOTUs were submitted for taxonomic analysis in the SILVA database (SINA, https://www.arb-silva.de/aligner/, October 2020, version 1.2.11 [26], SILVA reference database release 138.1) using default settings.

### 2.4. Morphological, Physiological and Chemotaxonomic Characterization

Strain 1933P^T^ was characterized using a polyphasic taxonomic approach and compared with the reference strain *Soehngenia saccharolytica* DSM 12858^T^ kindly provided by Dr. S.N. Parshina from the Research Center of Biotechnology of the Russian Academy of Sciences. Unless otherwise stated, both strains were cultivated in a liquid MM medium. This medium contained peptone (2 g L^−1^), yeast extract (0.2 g L^−1^), 1.5% NaCl for strain 1933P^T^, sucrose (2 g L^−1^), yeast extract (1.0 g L^−1^) and 0.1% (*w*/*v*) NaCl for strain DSM 12858^T^. Optimal temperature conditions were determined by growing the strain 1933P^T^ in liquid medium at 8, 13, 20, 25, 30, 35, 42, 45, 50, 55, and 60 °C. Salt tolerance tests for strains 1933P^T^ and DSM 12858^T^ were carried out in liquid medium supplemented with 0–6 % (*w*/*v*) (0, 0.1, 0.5, 1.0, 1.5, 2.0, 2.5, 3.0, 3.5, 4.0, 4.5, 5.0, 5.5, and 6.0 %) of NaCl at 35 °C. The pH range for growth of the isolate was tested in the media adjusted to pH 5.5, 6, 6.3, 6.7, 7.0, 7.5, 8.0, 8.5, and 9.0 with the addition of the appropriate buffer at optimal temperature (35 °C) and NaCl concentration (1.5% (*w*/*v*) NaCl) [27].

The cells’ sizes were measured on living cells using an Axio Imager.D1 epifluorescence microscope (Carl Zeiss, Oberkochen, Germany) with an Axio Cam HRc digital camera and Axio Vision computer software. The gram reaction and cell ultrastructure were studied as described previously [27]. Ultrathin sections were examined under a JEM-100C transmission electron microscope (JEOL, Tokyo, Japan) at an accelerating voltage of 80 kV. Biochemical and enzyme characteristics of strains 1933P^T^ and DSM 12858^T^ were determined by using API 50CH, API ZYM, and API 20E kits (bioMérieux, Marcy-l’Étoile, France) according to the manufacturer’s instructions, and incubated 7 days at 35 °C; to prevent oxygen access, mineral oil was added to each well. Catalase activity was determined by the standard method involving addition of 3% (*v*/*v*) H_2_O_2_ to concentrated cell suspensions. Oxidase activity was determined using the oxidase reagent (bioMérieux, France). Growth of the strain 1933P^T^ was additionally tested in MM medium with yeast extract (0.2 g L^−1^) and various substrates. The concentrations of sugars and biopolymers were 2 g L^−1^; those of organic acids and alcohols were 20 mM, and the H_2_ + CO_2_ mixture was tested at 80: 20 (*v*/*v*). Growth was monitored by the optical density (OD) at 660 nm. OD increases of <10, 10–50, and >50% obtained with the test substrates were scored as no utilization (−), weak utilization (W) and good utilization (+). Growth was registered for up to 14 days in three successive transfers. All tests were performed in duplicate. Strain 1933P^T^ was tested for its ability to use sulfate (20 mM), thiosulfate (15 mM), sulfite (15 mM), sulfur (5 g L^−1^), and nitrate (20 mM) as electron acceptors. Sulfide was measured by the colorimetric method with N,N-dimethyl-*p*-phenylenediamine in the modification by Trüper and Schlegel [28]; nitrite was determined using the Griess reagent. Fermentation products were analyzed by gas chromatography as described previously [29]. Nitrogen fixation was estimated by acetylene reduction assay in mineral medium with maltose (2.0 g L^−1^) and yeast extract (0.1 g L^−1^) under the N_2_ gas phase amended with 10% (*v*/*v*) acetylene. Ethylene production in each vial was quantified, using a gas chromatograph equipped with a flame ionization detector and a capillary column, as recommended [30].

The fatty acid composition was analyzed using a Maestro gas chromatograph–mass spectrometer (Interlab, Moscow, Russia). The cell biomass was dried with methanol and subjected to acidic methanolysis (1.2 M HCl/MeOH, 80 °C, 45 min) as described earlier [31]. The analysis of polar lipids of strains 1933P^T^ and *S. saccharolytica* DSM 12858^T^ was performed at the All-Russian Collection of Microorganisms according to the method described by Minnikin et al. [32]. Polar lipids were extracted from freeze-dried cells. The lipids were separated by two-dimensional TLC on Silica Gel 60F TLC-plates (Merck) using the following solvent systems: chloroform/methanol/water (65: 25: 4, by vol.) in the horizontal dimension and chloroform/acetic acid/methanol/water (80: 15: 12: 4, by vol.) in the vertical dimension. Total lipids were visualized by spraying with a 5 % (*w*/*v*) solution of phosphomolybdic acid in ethanol. Phospholipids were further characterized by spraying with ninhydrin (specific for amino groups), molybdenum blue (specific for phosphates) and α-naphthol (specific for glycolipids).

### 2.5. 16S rRNA Gene Sequencing and Phylogenetic Analysis

DNA was extracted from 1933P^T^ culture using the PowerSoil DNA Isolation Kit (MoBio, USA), according to the manufacturer’s recommendations. The 16S rRNA gene of the strain 1933P^T^ was amplified with the 27F and 1492R primers [33], and purified PCR products were sequenced with an ABI Prism 3730 DNA analyzer (Applied Biosystems, Foster City, CA, USA) using the Big Dye Terminator reagent kit, version 3.1. The 16S rRNA gene sequence analysis was performed using the EzBioCloud [34]. Phylogenetic analysis of the 16S rRNA gene sequences was carried out using the maximum-likelihood, neighbour-joining, and maximum-parsimony algorithms. The sequences were first aligned by MUSCLE [35], and the maximum-likelihood tree was inferred using the GTR + F + I + G4 model recommended by ModelFinder [36] in IQ-Tree [37]. Neighbour-joining and maximum-parsimony trees were reconstructed using the MEGA7 software package [38]. Bootstrap values were calculated from 1000 alternative trees.

### 2.6. Genome Analysis

The genome of the strain 1933P^T^ was sequenced and annotated as described previously [16]. Phylogenomic analysis of strain 1933P^T^ and members of the families *Tissierellaceae* and *Gottschalkiacecae* was conducted using a concatenated alignment of 120 single-copy phylogenetic marker genes obtained using the software GTDB-Tk version 1.0.2 [39]. A maximum likelihood phylogenomic tree was calculated using IQ-Tree [37] according to the model recommended by ModelFinder [36] and branch support was estimated using UFBoot2 [40]. Maximum parsimony and neighbour-joining trees were reconstructed using MPBoot [41] and MEGA7 [38], respectively. The pair-wise average nucleotide identity (ANI) and digital DNA–DNA hybridization (dDDH) values among 1933P^T^, *S. saccharolytica* DSM 12858^T^, and strains of other genera of the family *Tissierellaceae* based on their whole genomes were calculated using the ANI calculator (https://ani.jgi.doe.gov/html/calc.php?) [42] and the genome-to-genome distance calculator version 2.1 [43] with BLAST+ for genome alignments [44], respectively. Average amino acid identity (AAI) values were calculated using CompareM 0.0.23 (https://github.com/dparks1134/CompareM) with default blastp parameters (i.e., e-value ≤ 0.001, percent identity ≥ 30% and alignment length ≥ 70%). The pairwise percentage of conserved proteins (POCP) was calculated using the runPOCP.sh script [45,46], which was based on a previously published approach [47]. Eleven *Tissierellaceae* genomes were used for a pangenomic analysis. The analysis was done following the bioinformatic pipeline proposed [48] with the anvi’o program version 6.2 [49]. The genomes were organized based on the distribution of gene clusters using the MCL algorithm (distance: Euclidean; linkage: Ward). Functional genome annotations were performed using DRAM [50].

### 2.7. Nucleotide Sequence Accession Numbers

The GenBank/EMBL/DDBJ accession number of the 16S rRNA gene sequence of strain 1933P^T^ is MN698738.1. The GenBank/EMBL/DDBJ accession number of the genome of strain 1933P^T^ is SRIB00000000 (version SRIB01000000) [16]. The 16S rRNA gene library of the methanogenic enrichment was deposited to NCBI, project PRJNA673732, SRR12971726.

## 3. Results and Discussion

### 3.1. Phylogenetic Diversity of Prokaryotes in Methanogenic Enrichment

The methanogenic enrichment 1933, which was stored for 33 years at room temperature in the medium with methanol without transfer to fresh medium, was used for sequencing of the V4 hypervariable region of prokaryotic 16S rRNA genes. The resulting dataset upon filtration consisted of 35,373 reads assigned to *Bacteria* and 104 reads assigned to *Archaea*. A minority of archaea in methanogenic enrichment is likely due to death and lysis of methanogens over the 33 years period. Archaeal sequences (˂0.25% in the library) belonged to members of *Euryarchaeota* (genera *Methanosarcina* and *Methanomethylovorans*). Bacterial groups revealed in the enrichment were affiliated to the class *Deltaproteobacteria* (56.9% of sequences in the library) and to the phyla *Firmicutes* (26.3%), *Synergistetes* (9.8%), *Bacteroidetes* (4.7%), and *Actinobacteria* (1.1%). Minor components of the enrichment belonging to the phyla *Thermotogae*, *Spirochaetes*, and *Chloroflexi* were each responsible for ˂1% of the sequences in the library. At the species level the enrichment was dominated by a sulfate-reducing bacterium *Desulfovibrio aminophilus* (55.9% of sequences), fermenting bacterium *Soehngenia* sp. (26.1%), *Thermovirga* sp. (8.8%), *Petrimonas sulfuriphila* (3.2%), and *Proteiniphilum* sp. (0.8%). The fragments of their 16S rRNA genes had more than 99.6% similarity with the genes of respective bacteria.

In this enrichment only methanogenic archaea of the genera *Methanomethylovorans* and *Methanosarcina*, which were among strains able to grow on methanol, survived for 33 years [51,52]. *Desulfovibrio aminophilus* was detected in the enrichment, despite sulfate not being added into the medium. Sulfate may come from sulfur organic molecules, including amino acids and yeast extract. This bacterium is known to be capable of growing on amino acids, H_2_/CO_2_, formate, and ethanol as electron donors with sulfate as an electron acceptor, and fermented pyruvate, casamino acids, or peptone in the absence of sulfate in the medium [53]. A moderately thermophilic, anaerobic, amino acid-degrading bacterium *Thermovirga lienii* and a mesophilic fermentative sulfur-reducing bacterium *P. sulfuriphila* were detected in the enrichment. These bacteria were originally isolated from petroleum reservoirs and also could not use methanol for growth [54,55]. It is known that *Desulfovibrio aminophilus* and *Thermovirga* sp. are capable of growth by fermenting proteinaceous components of the biomass [53,54]. The products of fermentation of peptone, proteinaceous substrates, some amino acids, and a limited number of organic acids (but not sugars, fatty acids, or alcohols) by *T. lienii* include acetic and propionic acids, ethanol, H_2_, and CO_2_ [54]. The possible function of *Proteiniphilum* in the community is utilization of protein substrates and carbohydrates from cellular debris and production of acetate and CO_2_ [56,57]. Bacteria of the genus *Petrimonas* are capable of fermenting carbohydrates and some organic acids with production of acetate, hydrogen, and CO_2_ [55]. However, as a whole, metagenome analysis likely provides a picture of a part of the original methanogenic culture, without any possibility to associate a role to the detected microbes in an attempt to extrapolate a nutritional network that likely misses some components. Some of the bacteria and archaea that had a certain role in the crossfeeding relationship, are probably not present anymore, and their nucleic acids have been degraded a long time ago.

### 3.2. Phenotypic Characterization of Strain 1933P^T^

**Figure 1:**
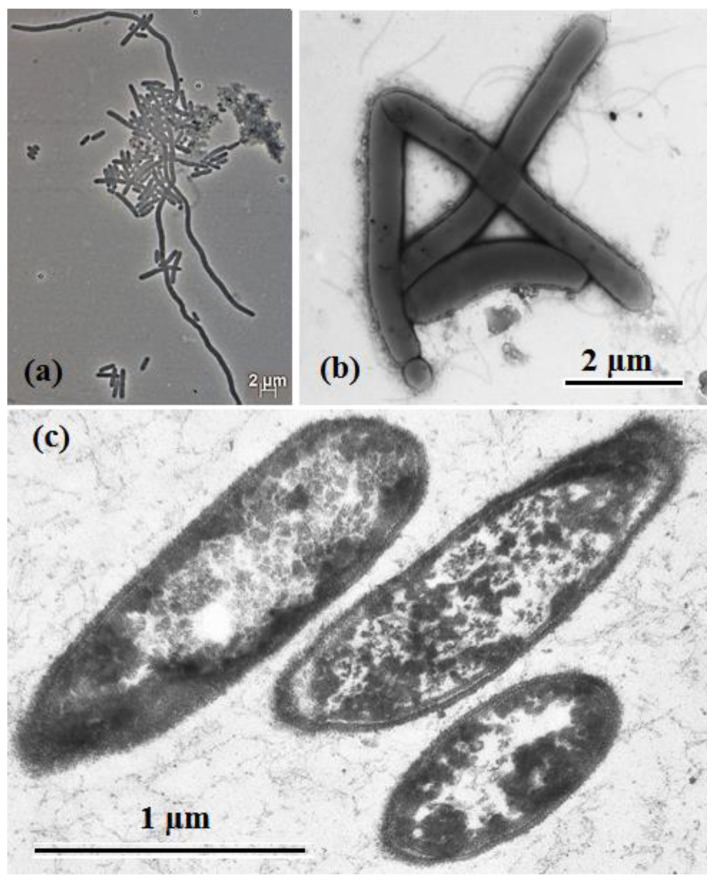
Phase contrast micrographs of cells: (**a**) transmission electron micrographs of negatively stained flagellated cells, (**b**) sections (**c**) of strain 1933P^T^ grown for 120 h in mineral medium (MM) with peptone and yeast extract at 35 °C.

Strain 1933P^T^ and its closely related species, *S. saccharolytica* DSM 12858^T^, were phenotypically characterized. Cells of the strain 1933P^T^ were 0.5 μm in width and 2–5 μm in length, gram-stain-positive, motile, peritrichously flagellated rods with rounded ends. Cell division was usually symmetric, but small cells and chains up to 150 μm in length were also visible in the culture (Figure 1a,b). On ultrathin sections the cells had a gram-positive cell wall structure (Figure 1c). Endospores occurred rarely and were terminal, round, and did not distend the mother cell. Strain 1933P^T^ grew at 13–55 °C (optimum 35 °C), at pH 6.7–8.0 (optimum pH 7.0) and with 0–3.0% (*w*/*v*) NaCl (optimum 0–2.0%) (Figure S1a–c). Strain *S. saccharolytica* DSM 12858^T^ grew in the presence of 0–2.0% (*w*/*v*) NaCl (optimum, 0–0.5% NaCl) (Figure S1d). Comparative morphological, physiological, and biochemical characteristics of strain 1933P^T^ and of phylogenetically related bacteria *S. saccharolytica* DSM 12858^T^ and *Gudongella oleilytica* W6^T^ [1] are summarized in Table 1.

Strain 1933P^T^ was an anaerobic bacterium. It did not grow in the medium without reductant and in non-reduced medium under 7% (*v*/*v*) air in the gas phase. Electron acceptors including nitrate, sulfate, and sulfite were not reduced by strain 1933P^T^ in the MM medium with yeast extract (2 g L^−1^), but sulfur and thiosulfate stimulated the growth of the strain and were reduced with production up to 90 mg H_2_S per liter. Nitrate was not reduced to nitrite. The major fermentation products of strain 1933P^T^ growing in MM medium with yeast extract (2 g L^−1^) were acetic acid, H_2_, and CO_2_, while minor amounts of ethanol, propionic, *n*-butyric and *iso*-valeric acids were formed. The major products of yeast extract fermentation by strain DSM 12858^T^ were acetic acid, ethanol, acetone, H_2_, and CO_2_, and minor amounts of propionic, *iso*-butyric and *n*-butyric acids were formed. Although the ability to fix dinitrogen was stated in the description of *S. saccharolytica*, acetylene assay experiments did not detect this ability in strains DSM 12858^T^ and 1933P^T^. Strain 1933P^T^ did not grow on benzaldehyde.

As determined using the API ZYM and API 20E tests (bioMérieux, France), strain 1933P^T^ had high activities of alkaline phosphatase and β-glucosidase, as well as weak activities of esterase-lipase (C8), acid phosphatase and naphthol-AS-BI-phosphohydrolase and was negative for indole production (Table S1). *S. saccharolytica* strain DSM 12858^T^ exhibited positive activity of acid phosphatase and naphthol-AS-BI-phosphohydrolase, was weakly positive on β-glucosidase and there was negative activity of alkaline phosphatase and esterase-lipase (C8). Both strains had negative activities of esterase (C4), lipase (C14), leucine arylamidase, valine arylamidase, cysteine arylamidase, trypsin, α-chymotrypsin, α-galactosidase, β-galactosidase, β-glucuronidase, α-glucosidase, N-acetyl-β-glucosaminidase, α-mannosidase, urease, tryptophan deaminase, lysine decarboxylase, ornithine decarboxylase, α-fucosidase, and citrate utilization.

In growth experiments strain 1933P^T^ fermented yeast extract, peptone, tryptone, mannose, but could not grow on formate, acetate, propionate, pyruvate, casein hydrolysate, ethanol, methanol, propanol, leucine, glycine, alanine, and cannot produce acid from fructose, glucose, sucrose, trehalose, cellobiose, raffinose, arabinose, cellulose, starch, xylan, mannitol, or glycerol. In our experiments using the API 50CH kit, strains 1933P^T^ and DSM 12858^T^ were negative for acid production from d-adonitol, d-arabinose, d-arabitol, d-fucose, d-lactose, d-melezitose, d-sorbitol, dulcitol, erythritol, l-rhamnose, l-sorbose, l-xylose, methyl α-d-mannopyranoside, and xylitol. Strain 1933P^T^ did not produce acid from amygdalin, l-arabinose, d-xylose, d-galactose, inositol, methyl α-d-glucopyranoside, d-melibiose, gentiobiose, d-tagatose, and l-arabitol; these tests were positive for strain DSM 12858^T^.

The major fatty acids of strain 1933P^T^ were C_14:0_ (36.7%), C_16:0_ (29.7%), and *iso*-C_15:0_ (8.3%), while *S. saccharolytica* DSM 12858^T^ was mainly composed of C_16:0_ (41.4%), C_18:1_ (21.1%), and C_16:1_ (20.5%) (Table S2). Because of the problems recovering enough biomass of strain 1933P^T^, we could not reliably determine the polar lipid profile of this strain (Figure S2a, Table 1). Strain DSM 12858^T^ had unidentified phosphoglycolipids, phospholipids, diphosphoglycolipids, and lipids (Figure S2b), which was consistent with results of Wu et al. [1].

### 3.3. Phylogenetic Analysis of 16S rRNA Gene Sequences

**Figure 2:**
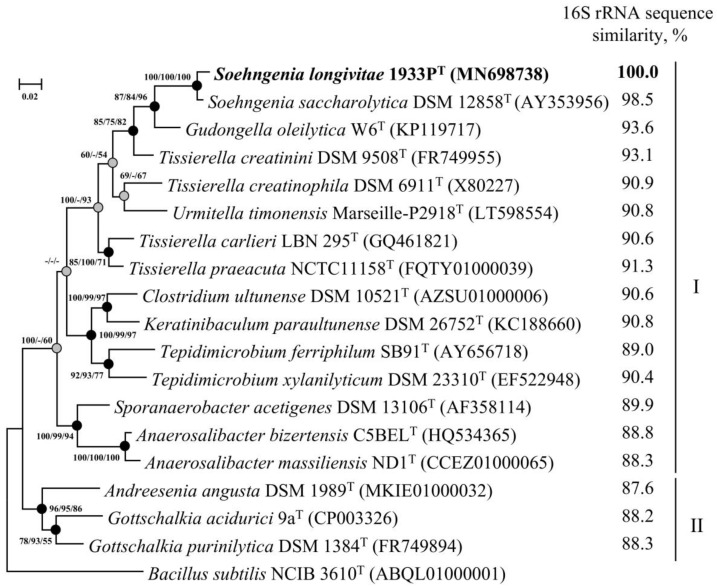
Maximum-likelihood phylogenetic tree based on 16S rRNA gene sequences (1498 nucleotide sites) reconstructed with the evolutionary model GTR + F + I + G4, showing the position of strain 1933P^T^ and closely related members of the families *Tissierellaceae* (I) and *Gottschalkiaceae* (II). Grey circles indicate that the corresponding nodes were recovered in the tree that was reconstructed based on the maximum parsimony algorithm; black circles indicate that the corresponding nodes were also recovered based on the neighbour-joining and maximum-parsimony algorithms. Bootstrap values (>50%) are listed as percentages at the branching points. Scale bar, 0.02 substitutions per nucleotide position. The 16S rRNA gene sequence similarity refers to strain 1933P^T^ compared to the rest of the species. The tree was rooted using *Bacillus subtilis* NCIB 3610^T^ as the outgroup. GenBank accession numbers for 16S rRNA genes are indicated in brackets.

The complete 1476-bp 16S rRNA gene sequence of strain 1933P^T^ was obtained using PCR. The V4 hypervariable region of the sequenced 16S rRNA gene was identical to zOTU of *Soehngenia* sp., determined from methanogenic enrichment 1933. On phylogenetic trees inferred from the maximum-likelihood, neighbour-joining and maximum-parsimony algorithms, strain 1933P^T^ formed a distinct lineage in the clade with *S. saccharolytica* DSM 12858^T^ separated from strains of the other genera of the family *Tissierellaceae*, indicating that strain 1933P^T^ is a member of the genus *Soehngenia* (Figure 2). The 16S rRNA gene sequences of strains 1933P^T^ and DSM 12858^T^ had 98.5% similarity, which was below the threshold accepted for species demarcation [17] and suggested affiliation of the strain 1933P^T^ to a new species.

### 3.4. Whole Genome Sequencing and Phylogenomic Analyses

The final assembled 1,917,091-bp-long genome of strain 1933P^T^ was comprised of 33 scaffolds, with an N_50_ value of 132,646 bp, G+C content of 31.9%, and coverage of 630×. The genome contained 1853 genes, of which 1789 were protein-coding sequences, 23 were pseudogenes, and 41 coded RNAs. Functional annotation of the genome was performed with a RAST server [58,59], via the RASTtk pipeline with the default settings [60]; it revealed that 150 of the genes were associated with protein metabolism, 106 genes, with metabolism of amino acids and derivatives, 101 genes, with carbohydrate metabolism, and 40 genes, with metabolism of cofactors, vitamins, and pigments (Figure S3). The genome of strain 1933P^T^ was compared with that of *S. saccharolytica* DSM 12858^T^. The genome of strain DSM 12858^T^ was obtained from the JGI IMG database (accession number 2571042347) [61]. General properties of the genomes of both strains are summarized in Table S3.

**Figure 3:**
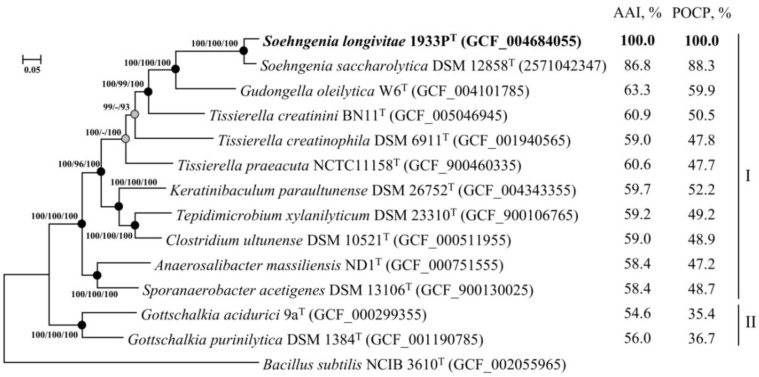
Maximum-likelihood phylogenetic tree derived from concatenated 120 single copy marker proteins showing the position of strain 1933P^T^ in relation to taxonomically characterized members of the families *Tissierellaceae* (I) and *Gottschalkiacecae* (II). Phylogenetic analysis was performed with an LG + F + I + G4 model based on 34,747 amino acid positions. Scale bar, 0.05 amino acid substitutions per site. The tree was rooted using *Bacillus subtilis* NCIB 3610^T^ as the outgroup. Accession numbers for the genomes are indicated in brackets. The average amino acid identity (AAI) and pairwise percentage of conserved proteins (POCP) values refer to strain 1933P^T^ compared to the rest of the species.

On the phylogenomic tree, strain 1933P^T^ formed a branch with *S. saccharolytica* DSM 12858^T^ (Figure 3). The ANI and dDDH values of 83.5% and 27.0%, respectively, to the genome of the closest species *S. saccharolytica* DSM 12858T were below the species cutoff (95–96% for ANI and 70% for dDDH) [62], which indicates that the strain 1933P^T^ belongs to a new species (Table S4). Comparison of strain 1933P^T^ with *S. saccharolytica* DSM 12858^T^ yielded AAI and POCP values of 86.8% and 88.3%, respectively, which were higher than the proposed genus thresholds of 65% for AAI [63] and 50% for the POCP [47] values. Thus, according to the results of genome-based phylogenomic analysis, strain 1933P^T^ may be classified as a novel species within the genus *Soehngenia*, for which the name *Soehngenia longivitae* sp. nov. is proposed.

**Figure 4:**
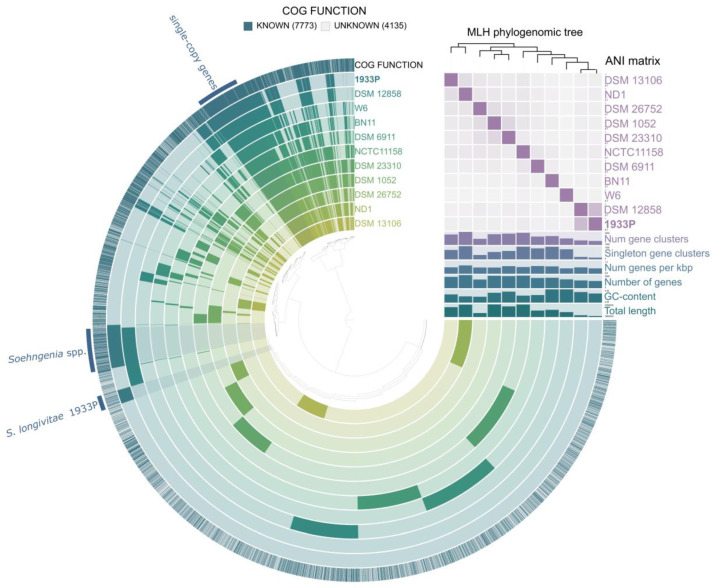
Pangenome analysis of eleven genomes of *Tissierellaceae* was calculated with Anvi’o version 6.2. The dendrogram at the center represents the relationship between the 11,908 gene clusters (29,926 genes) found in the analyzed genomes. Dark regions in colored circles represent the genes found in that area for each genome. The average nucleotide identity (ANI) heatmap in purple squares varies between 70 and 100%. The phylogenomic tree was reconstructed using the single copy genes.

A total of nine genomes were used for the pangenomic analysis of the *Tissierellaceae* species. The pangenome of *Tissierellaceae* comprised 29,296 genes in 11,908 gene clusters, and 482 in the core genome (Figure 4). Functional analysis of the core genome proteins revealed that 222 proteins were associated with processing of the genetic information, 31 with nucleotide metabolism, and 30 with carbohydrate metabolism. All *Tissierellaceae* genomes contained genes for glycolysis, pyruvate oxidation, and phosphate acetyltransferase-acetate kinase pathway. All genomes lacked electron transport chain genes (Figure S4). Strain 1933P^T^ and *S. saccharolytica* DSM 12858^T^ had 417 shared gene clusters that are not present among other members of *Tissierellaceae*, and 222 of them were functionally annotated. Shared genes were involved in genetic (57) and environmental (30) information processing, signaling and cellular processes (21), carbohydrates (18), amino acids (12), and vitamins/cofactors (11) metabolisms. *Soehngenia* genomes carried 22 ABC transporters, which were responsible for iron (III) (*afuABC*), molybdate (*modABC*), raffinose/stachyose/melibiose (*msnEFG*), sugars (*chvE*, *gguAB*), phosphate (*pstACS*), oligopeptide peptides (*oppAC*), and branched-chain amino acids (*livGHKM*) acquisition. The genes responsible for fixation of molecular nitrogen were absent in both strains, which correlated with the results of our in vitro tests.

The genome of strain 1933P^T^ contained 145 unique gene clusters, but only 36 of them had a predicted function. Among them, the genes responsible for lycopene biosynthesis (phytoene desaturase *crtI*, 15-cis-phytoene synthase *crtB*, and phytol kinase VTE5) were found. Unlike the *S. saccharolytica* DSM 12858^T^ genome, the 1933P^T^ strain lacked ribose/D-xylose transporters (*rbsABC*).

Genome analysis shows that the metabolism of bacteria of the genus *Soehngenia* in the methanol-degrading methanogenic enrichment was probably based on fermentation of proteins and amino acids. Genome sizes for *Soehngenia* sp. 1933P^T^ and *S. saccharolytica* DSM 12858^T^ (accession number in the JGI IMG database is 2571042347) were 1.92 and 2.00 Mb, respectively (Table S3), which indicated the low catabolic potential of these bacteria. Growth of these rarely occurring bacteria is probably dependent on activity of microorganisms of the previous trophic level, which provide them with available substrates, and is possibly based on metabiotic and syntrophic interactions. It was suggested that rare taxa may represent a reservoir of genetic diversity that actively responds to environmental change [64,65,66]. Oilfields have low levels of water and mass exchange and contain oil, gaseous hydrocarbons, and products of oil oxidation as the major sources of organic matter. Microbial populations of these habitats, including rare species, are adapted to the conditions, as was demonstrated by analysis of the methanogenic enrichment. The 1933P^T^ strain was the only component of the community that could survive adverse conditions due to spore formation. Elucidation of the mechanisms for dormancy preservation by other members of a long-stored methanogenic enrichment requires further investigation.

### 3.5. Description of Soehngenia Longivitae sp. nov

*Soehngenia longivitae* (lon.gi.vi’tae. L. masc. adj. *longus*, long; L. fem. n. *vita* life; N.L. gen. n. *longivitae* of long life).

The description is based on a single strain: gram-stain-positive rods, 0.5 μm in diameter and 2–5 μm long, motile by peritrichous flagella, with gram-positive structure of the cell wall. Endospores occur rarely and are terminal, round, and do not distend the mother cell. Catalase- and oxidase-negative. They are chemoorganoheterotrophic anaerobes; fermentative growth is observed with proteinaceous substrates. They are mesophilic and neutrophilic. The temperature range is 13 to 55 °C (optimum, 35 °C), the pH range is 6.7–8.0 (optimum, pH 7.0) and the NaCl range is 0–3.0% (*w*/*v*) (optimum, 0–2.0% NaCl). Sulfate, sulfite, and nitrate are not used as electron acceptors. Yeast extract, peptone, tryptone, and mannose are fermented. Acetic acid, butyric acid, H_2_, and CO_2_ are the major products of yeast extract fermentation. It cannot grow with formate, acetate, propionate, pyruvate, casein hydrolysate, ethanol, methanol, propanol, fructose, glucose, sucrose, trehalose, cellobiose, raffinose, arabinose, cellulose, starch, xylan, mannitol, and glycerol. The main cellular fatty acids were C_14:0_, C_16:0_, and *iso*-C_15:0_.

The type strain is 1933P^T^ (= VKM B-3382^T^ = KCTC 15984^T^), isolated from a methanogenic enrichment obtained from a production water sample from the Binagady oilfield (Baku city, Republic of Azerbaijan). The G + C content of the genome of the strain is 31.9 mol%, its approximate size is 1.917 Mbp. The GenBank/EMBL/DDBJ accession numbers of the 16S rRNA gene and genome sequences of strain 1933P^T^ are MN698738.1 and SRIB00000000 (version SRIB01000000), respectively.

Emended description of the genus *Soehngenia* Parshina et al. 2003.

The description of the genus *Soehngenia* is as that given by Parshina et al. 2003, with the following modification. They are anaerobic or facultatively anaerobic, and saccharolytic and proteolytic. The genomic G + C content is about 32–33%. The size of genomes varies by around 2.0 Mbp. Members of this genus form a monophyletic clade in phylogenetic trees based on concatenated sequences for different large datasets of proteins and also in a tree based on 16S rRNA gene sequences. The type species is *Soehngenia saccharolytica* BOR-Y^T^ (= DSM 12858^T^ = ATCC BAA-502^T^). The genomic G + C content of *S. saccharolytica* DSM 12858^T^ is 32.9%. The GenBank/EMBL/DDBJ accession number of the 16S rRNA gene and the JGI IMG accession number of the genome sequence of strain DSM 12858^T^ are AY353956 and 2571042347, respectively.

## 4. Conclusions

A long-stored methanogenic enrichment obtained from a petroleum reservoir in Azerbaijan was studied by 16S rRNA gene sequence analysis and its bacterial and archaeal diversity was revealed. From this enrichment a new mesophilic fermentative bacterium, strain 1933P^T^, was isolated in pure culture. The taxonomic study including a phylogenetic 16S rRNA gene sequence and genome analyses, chemotaxonomic, and phenotypic studies showed that this strain represented a new species within the genus *Soehngenia*, for which we propose the name *Soehngenia longivitae* sp. nov. Genome analysis of the novel strain revealed its potential in the destruction of proteinaceous components of the biomass in the enrichment and the dominance of genes involved in protein metabolism.

## Figures and Tables

**Table 1:** Characteristics differentiating strain 1933P^T^, *S. saccharolytica* DSM 12858^T^ [3] *, and *Gudongella oleilytica* [1].

Characteristics	1933P^T^	*S. saccharolytica* DSM 12858^T^	*Gudongella oleilytica* W6^T^
Cell size, µM	0.5 × 2–5	0.5–0.7 × 2–11	2–4 × 9–21
Spore formation	+	+	–
Temperature range (optimum), °C	13–55 (35)	15–40 (30–37)	20–45 (40)
pH range (optimum)	6.7–8.0 (7.0)	6.5–7.5 (7.0)	6.5–9.0 (7.5)
NaCl range (optimum), % *w*/*v*	0–5.0 (0–2.0)	0–2.0 (0) *	0–3.5 (0)
Genomic G + C content, %	31.9	32.94	42.4
Genome size (Mb)	1.92	2.0	2.36
Utilization of			
Pyruvate	−	+	W
Arabinose	−	+	W
Cellobiose	−	+	W
Galactose	−	+	−
Glucose	−	+	−
Fructose	−	+	−
Lactose	−	+	−
Maltose	+	+	−
Mannose	W	+	−
Mannitol	−	+	
Ribose	−	+	−
Sucrose	−	+	−
Raffinose	−		−
Xylose	−	+	−
Xylan	–	+	ND
Peptone	+	−	+
Methionine	−	−	+
Serine	−	+	ND
Hydrolysis of gelatin	W	−	ND
Molecular nitrogen fixation	−	– *	+
The main products of the yeast extract fermentation	Acetate, H_2_, CO_2_	Acetate, H_2_, CO_2_	ND
Electron acceptors:			
Elemental sulfur	+	−	ND
Sulfite	−	+	ND
Major fatty acids	C_14:0_, C_16:0_, iso-C_15:0_	C_16:0_, C_18:1_, C_16:1_ *	iso-C_15:0_, C_14:0_, C_16:0_, iso-C_13:0_
Major polar lipids	ND	PGL, PL, L, DPG *	L, AL
Isolation source	Methanogenic enrichment from oilfield	Anaerobic-digester sludge	Oily sludge from oilfield

* Data from this study. ND—no data. Designations: +, positive; −, negative; (W), weakly positive. All strains were rod-shaped, gram-stain-positive, utilized yeast extract, were negative for growth on formate, acetate, and propionate, and could not use sulfate as an electron acceptor.

## Supplementary Materials

The following are available online at https://www.mdpi.com/2076-2607/8/12/1967/s1. Figure S1: Growth profiles of strain 1933P^T^ at various temperatures (a), pH (b) and NaCl concentration (*w*/*v*, %) (c), and of *S. saccharolytica* strain DSM 12858^T^ (d) at various NaCl concentrations (*w*/*v*, %). Figure S2: Thin layer chromatogram of polar lipids extracts from strain 1933P^T^ (a) and *S. saccharolytica* DSM 12858^T^ (b). Designations: GL—glycolipids; PGL—phosphoglycolipids, PL—phospholipids; L—lipids; DPG—diphosphoglycolipids. Figure S3: Subsystems of strain 1933PT based on the SEED database. Figure S4: Heatmap profile showing the abundance of functional genes detected within the *Tissierellaceae* genomes. Heatmaps were automatically generated by DRAM. Sections of the heatmap are ordered to highlight information about pathway completion and ETC subunit completion (A). Boxes colored by presence/absence in (B) represent 1–2 genes necessary to carry out a particular process. Table S1: Comparison of enzymatic activities of strains 1933P^T^ and *S. saccharolytica* DSM 12858^T^ determined by the api^®^ZYM test (bioMérieux, France). Designations: +, positive; –, negative; W, weakly positive. Table S2: Fatty acid compositions of strain 1933P^T^ and type strain of *Soehngenia saccharolytica* DSM 12858^T^. Table S3: General properties and relationship of the genomes of strain 1933P^T^ and its closely related species *S. saccharolytica* DSM 12858^T^. Table S4: AAI and POCP values of strain 1933P^T^ and other related members of the families *Tissierellaceae* and *Gottschalkiacecae*.
