# Supplementary material for: Soehngenia longivitae sp. nov., a Fermenting Bacterium Isolated from a Petroleum Reservoir in Azerbaijan, and Emended Description of the Genus Soehngenia
Source: Microorganisms. 2020 Dec 11;8(12):1967. doi: 10.3390/microorganisms8121967 (PMC7763609; doi:10.3390/microorganisms8121967)
Supplement: Supplementary file 1 [file microorganisms-08-01967-s001.pdf]

Supplementary Material

Soehngenia longivitae sp. nov., a fermenting bacterium isolated from a petroleum reservoir in Azerbaijan, and emended description of the genus *Soehngenia*

Tamara N. Nazina [1,*], Salimat K. Bidzhieva [1], Denis S. Grouzdev [2], Diyana S. Sokolova [1], Tatyana P. Tourova [1], Sofiya N. Parshina [1], Alexander N. Avtukh [3], Andrey B. Poltaraus [4] and Azhdar K. Talybly [5]

[1] Winogradsky Institute of Microbiology, Research Center of Biotechnology, Russian Academy of Sciences, 117312 Moscow, Russia; salima.bidjieva@gmail.com (S.K.B.); sokolovadiyana@gmail.com (D.S.S.); tptour@rambler.ru (T.P.T.); sonjaparshina@mail.ru (S.N.P.); nazina@inmi.ru (T.N.N.)
[2] SciBear LLC, 13617 Tallinn, Estonia; denisgrouzdev@gmail.com (D.S.G.)
[3] Skryabin Institute of Biochemistry and Physiology of Microorganisms, Pushchino Scientific Center for Biological Research of the Russian Academy of Sciences, Pushchino, Moscow region 142290, Russia; avtukh@rambler.ru (A.N.A.)
[4] Engelhardt Institute of Molecular Biology, Russian Academy of Sciences, 119991 Moscow, Russia; abpolt@gmail.com (A.B.P.)
[5] Institute of Microbiology of the National Academy of Sciences of Azerbaijan, AZ1073 Baku, Azerbaijan ajdartalibli@mail.ru (AKT)

* Correspondence: nazina@inmi.ru; Tel.: +7-499-135-0341 (T.N.N.)

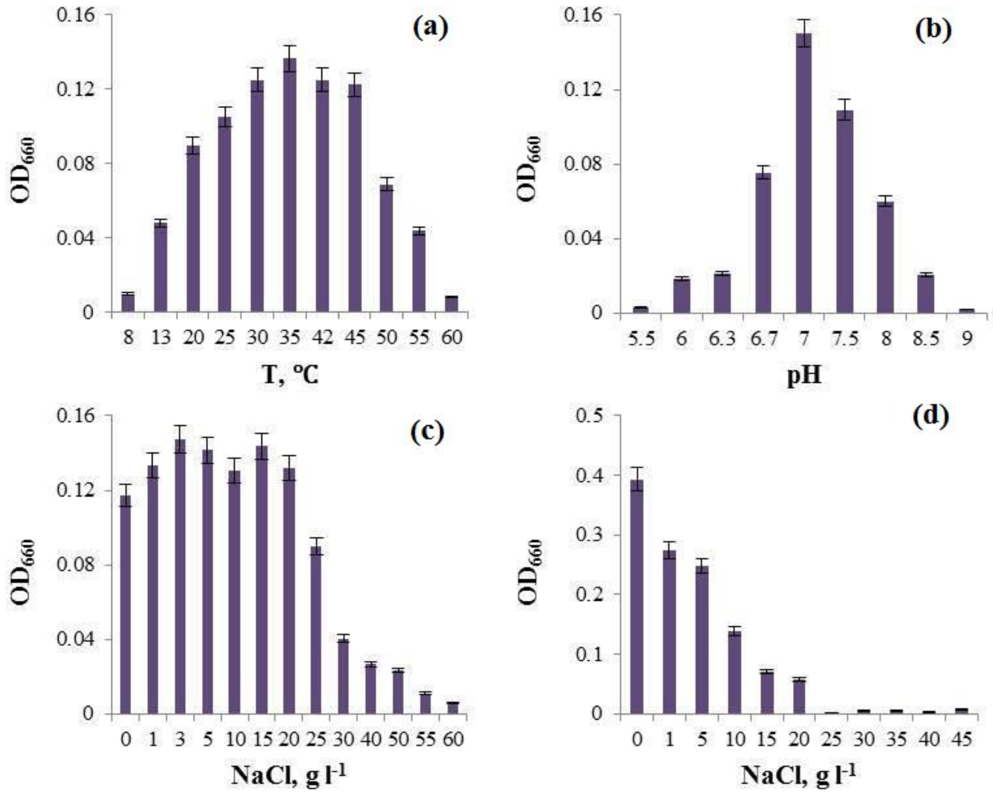

Figure S1: Growth profiles of strain 1933P^{T} at various temperatures (a), pH (b) and NaCl concentration (w/v, %) (c), and of *S. saccharolytica* strain DSM 12858^{T} (d) at various NaCl concentration (w/v, %).

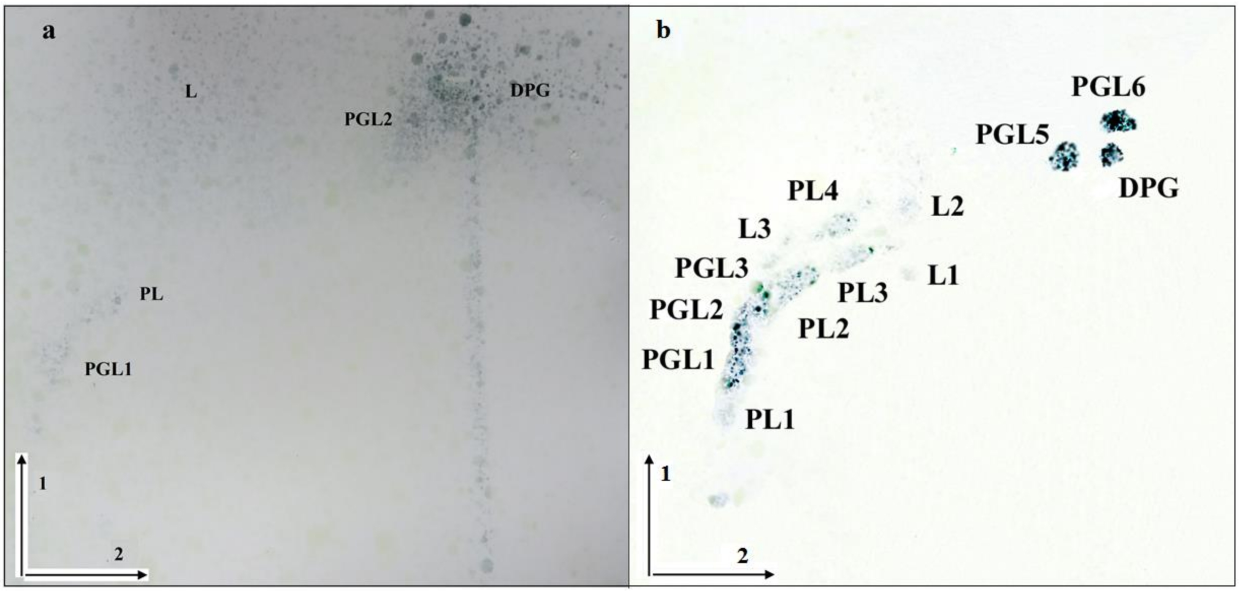

Figure S2: Thin layer chromatogram of polar lipids extracts from strain 1933P^{T} (a) and *S. saccharolytica* DSM 12858^{T} (b). Designations: GL – glycolipids; PGL – phosphoglycolipids, PL – phospholipids; L – lipids; DPG – diphosphoglycolipids.

.

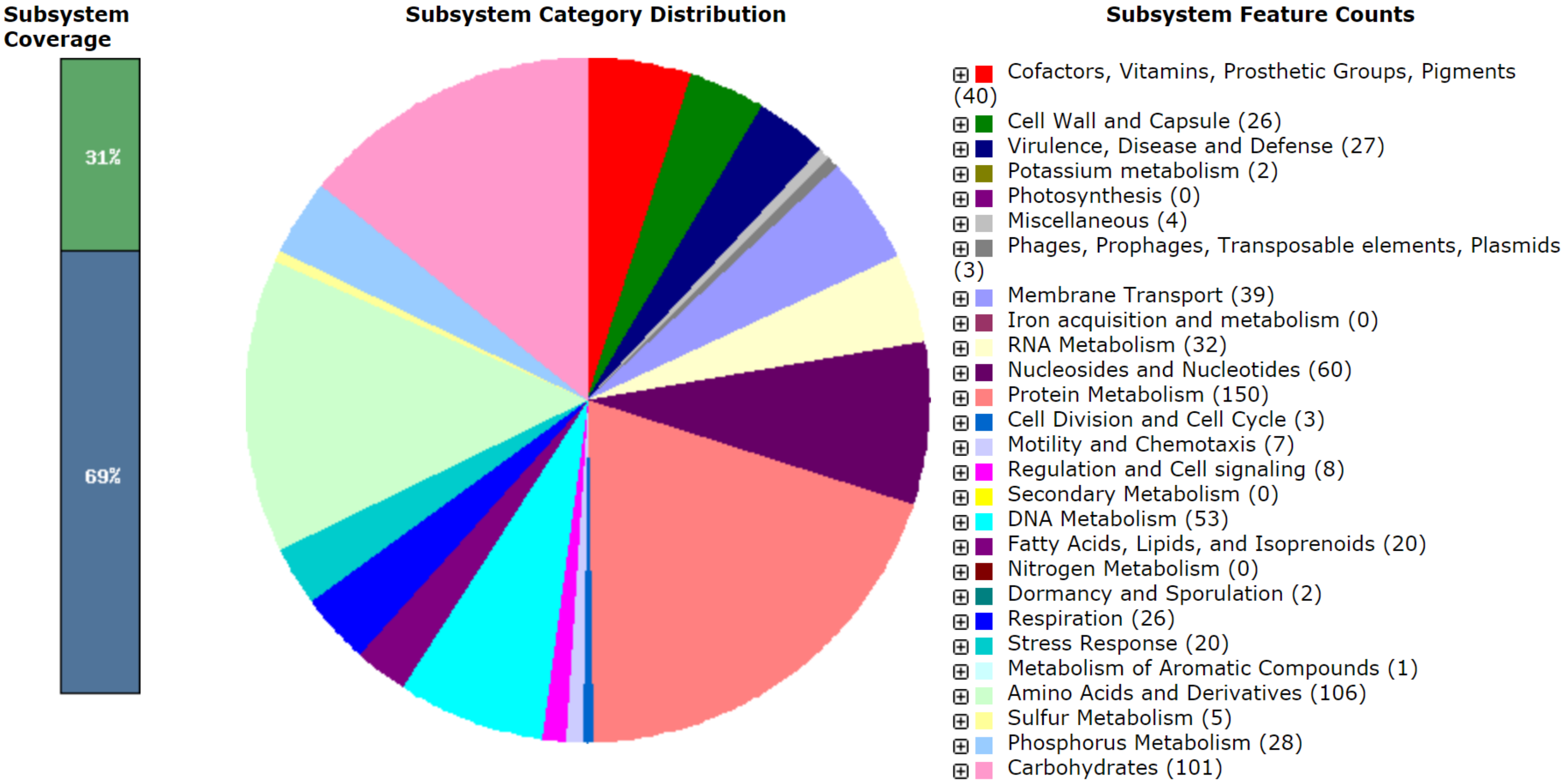

Figure S3: Subsystems of strain 1933P^{T} based on SEED database.

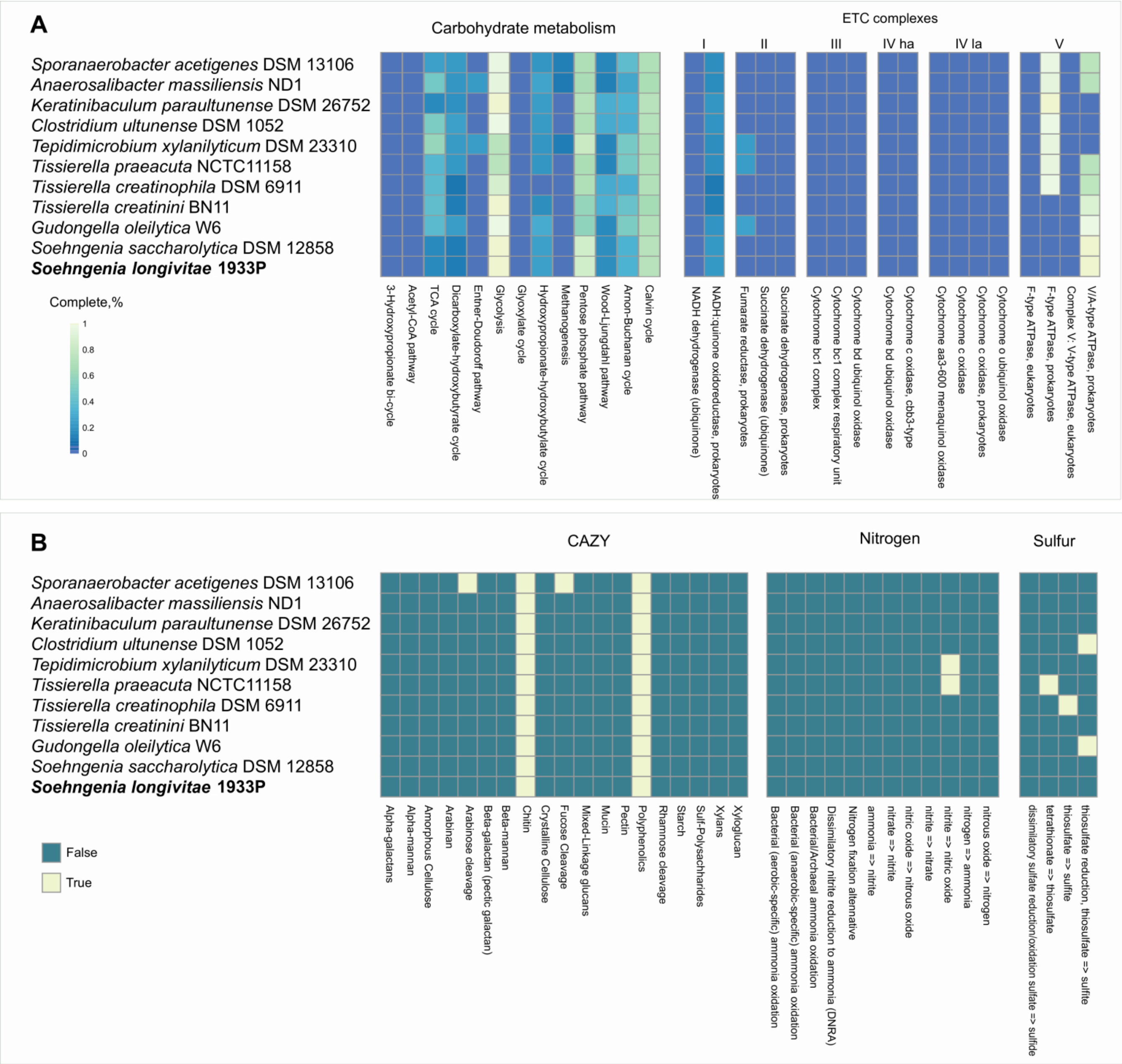
A
Carbohydrate metabolism
ETC complexes
I
II
III
IV ha
IV la
V
Sporanaerobacter acetigenes DSM 13106
Anaerosalibacter massiliensis ND1
Keratinibaculum paraultunense DSM 26752
Clostridium ultunense DSM 1052
Tepidimicrobium xylanilyticum DSM 23310
Tissierella praeacuta NCTC11158
Tissierella creatinophila DSM 6911
Tissierella creatinini BN11
Gudongella oleilytica W6
Soehngenia saccharolytica DSM 12858
Soehngenia longivitae 1933P
Complete,%
1
0.8
0.6
0.4
0.2
0
3-Hydroxypropionate bi-cycle
Acetyl-CoA pathway
TCA cycle
Dicarboxylate-hydroxybutyrate cycle
Entner-Doudoroff pathway
Glycolysis
Glyoxylate cycle
Hydroxypropionate-hydroxybutylate cycle
Methanogenesis
Pentose phosphate pathway
Wood-Ljungdahl pathway
Arnon-Buchanan cycle
Calvin cycle
NADH dehydrogenase (ubiquinone)
NADH:quinone oxidoreductase, prokaryotes
Fumarate reductase, prokaryotes
Succinate dehydrogenase (ubiquinone)
Succinate dehydrogenase, prokaryotes
Cytochrome bc1 complex
Cytochrome bc1 complex respiratory unit
Cytochrome bd ubiquinol oxidase
Cytochrome bd ubiquinol oxidase
Cytochrome c oxidase, cbb3-type
Cytochrome aa3-600 menaquinol oxidase
Cytochrome c oxidase
Cytochrome c oxidase, prokaryotes
Cytochrome o ubiquinol oxidase
F-type ATPase, eukaryotes
F-type ATPase, prokaryotes
Complex V: V-type ATPase, eukaryotes
V/A-type ATPase, prokaryotes
B
CAZY
Nitrogen
Sulfur
Sporanaerobacter acetigenes DSM 13106
Anaerosalibacter massiliensis ND1
Keratinibaculum paraultunense DSM 26752
Clostridium ultunense DSM 1052
Tepidimicrobium xylanilyticum DSM 23310
Tissierella praeacuta NCTC11158
Tissierella creatinophila DSM 6911
Tissierella creatinini BN11
Gudongella oleilytica W6
Soehngenia saccharolytica DSM 12858
Soehngenia longivitae 1933P
False
True
Alpha-galactans
Alpha-mannan
Amorphous Cellulose
Arabinan
Arabinose cleavage
Beta-galactan (pectic galactan)
Beta-mannan
Chitin
Crystalline Cellulose
Fucose Cleavage
Mixed-Linkage glucans
Mucin
Pectin
Polyphenolics
Rhamnose cleavage
Starch
Sulf-Polysaccharides
Xylans
Xyloglucan
Bacterial (aerobic-specific) ammonia oxidation
Bacterial (anaerobic-specific) ammonia oxidation
Bacterial/Archaeal ammonia oxidation
Dissimilatory nitrite reduction to ammonia (DNRA)
Nitrogen fixation alternative
ammonia => nitrite
nitrate => nitrite
nitric oxide => nitrous oxide
nitrite => nitrate
nitrite => nitric oxide
nitrogen => ammonia
nitrous oxide => nitrogen
dissimilatory sulfate reduction/oxidation sulfate => sulfide
tetrathionate => thiosulfate
thiosulfate => sulfite
thiosulfate reduction, thiosulfate => sulfite

Figure S4: Heatmap profile showing the abundance of functional genes detected within the *Tissierellaceae* genomes. Heatmaps were automatically generated by DRAM. Sections of the heatmap are ordered to highlight information about pathway completion and ETC subunit completion (A). Boxes colored by presence/absence in (B) represent 1-2 genes necessary to carry out a particular process.

Table S1: Comparison of enzymatic activities of strains 1933P^T and *S. saccharolytica* DSM 12858^T determined by the api®ZYM test (bioMérieux, France). +, positive; –, negative; W, weakly positive.

**Enzyme**	**1933P^T**	**DSM 12858^T**
Alkaline phosphatase	+	–
Esterase (C4)	–	–
Esterase Lipase (C8)	W	–
Lipase (C14)	–	–
Leucine arylamidase	–	–
Valine arylamidase	–	–
Cystine arylamidase	–	–
Trypsin	–	–
α-Chymotrypsin	–	–
Acid phosphatase	W	+
Naphthol-AS-BI-phosphohydrolase	W	+
α-Galactosidase	–	–
ß-Galactosidase	–	–
ß-Glucuronidase	–	–
α-Glucosidase	–	–
ß-Glucosidase	+	W
N-acetyl-ß-glucosaminidase	–	–
α-Mannosidase	–	–
α-Fucosidase	–	–

Table S2: Fatty acid compositions* of strain 1933P^T and type strain of *Soehngenia saccharolytica* DSM 12858^T.

**Fatty acid**	**1933P^T**	**DSM 12858^T**
$C_{11:0}$	0.4	
iso-$C_{12:0}$	0.3	
$C_{12:0}$	2.0	0.4
iso-$C_{13:0}$	1.9	
$C_{13:0}$	**6.3**	
iso-$C_{14:0}$	1.0	
$C_{14:1}$		0.4
$C_{14:0}$	**36.7**	**9.5**
iso-$C_{15:0}$	**8.3**	1.3
$C_{15:0}$	0.6	0.4
iso-$C_{16:0}$	0.2	
$C_{16:1}$	**5.1**	**20.5**
$C_{16:0}$	**29.7**	**41.4**
iso-$C_{17:0}$	0.3	
$C_{17:1}$		0.4
anteiso-$C_{17:0}$	0.2	
$C_{17:0}$	0.2	
$C_{18:1}$	3.9	**21.1**
$C_{18:0}$	2.3	4.0
Other	0.6	0.6
Total	100.0	100.0

*The values are percentages (w/w) of total fatty acids. Both strains were cultivated in a liquid MM medium for 7 days at 30 °C. This medium contained peptone (2 g l^{-1}), yeast extract (0.2 g l^{-1}), and 1.5% NaCl for strain 1933P^T, and sucrose (2 g l^{-1}), yeast extract (1.0 g l^{-1}) and 0.1% (w/v) NaCl for strain DSM 12858^T. Dominant fatty acids are indicated in bold.

Table S3: General properties and relationship of the genomes of strain 1933P^T and its closely related species *S. saccharolytica* DSM 12858^T.

**Attribute**	***S. longivitae* 1933P^T (GCF_004684055)**		***S. saccharolytica* DSM 12858^T (2571042347)**	
	Value	% of Total	Value	% of Total
Genome size (bp)	1,917,091	100.00	2,002,213	100.00
DNA coding (bp)	1,757,471	91.67	1,828,948	91.35
DNA G+C (bp)	610,836	31.86	659,599	32.94
DNA scaffolds	33	100.00	72	100.00
Total genes	1,853	100.00	2,029	100.00
Protein coding genes	1,789	96.55	1,953	96.25
RNA genes	41	2.21	76	0.30
Genes with function prediction	1,633	88.13	1,643	80.98
Protein coding genes with enzymes	601	32.43	622	30.66
Protein coding genes connected to KEGG pathways	657	35.46	679	33.46
Genes assigned to COGs	1,618	87.32	1,441	71.02
Genes with Pfam domains	1,622	87.53	1,712	84.38
Genes with signal peptides	51	2.75	72	3.55
Genes with transmembrane helices	431	23.26	448	22.08

Table S4: AAI and POCP values of strain 1933P^{T} and other related members of the families *Tissierellaceae* and *Gottschalkiaceae*

AAI \ POCP	Organism	1	2	3	4	5	6	7	8	9	10	11	12	13
1	***Soehngenia longivitae* 1933P^{T} (GCF_004684055)**	100	88.3	59.9	47.2	48.9	52.2	48.7	49.2	50.5	47.8	47.7	35.4	36.7
2	*Soehngenia saccharolytica* DSM 12858^{T} (2571042347)	86.8	100	59.5	46.4	49.7	52.7	48.4	49.6	50.1	46.8	46.8	35.1	36.5
3	*Gudongella oleilytica* W6^{T} (GCF_004101785)	63.3	63.1	100	40.3	46.8	45.6	43.0	44.6	52.0	45.5	46.7	33.5	34.5
4	*Anaerosalibacter massiliensis* ND1^{T} (GCF_000751555)	58.4	57.9	58.3	100	58.1	57.5	61.6	58.3	42.6	43.9	52.3	43.4	46.9
5	*Clostridium ultunense* DSM 1052^{T} (GCF_000511955)	59.0	59.2	59.9	65.1	100	63.6	57.9	67.4	48.2	45.9	56.2	41.8	43.5
6	*Keratinibaculum paraultunense* DSM 26752^{T} (GCF_004343355)	59.7	60.3	59.5	65.5	72.5	100	62.2	61.9	49.3	47.4	55.8	44.0	43.8
7	*Sporanaerobacter acetigenes* DSM 13106^{T} (GCF_900130025)	58.4	58.5	58.6	69.6	65.2	65.8	100	58.4	45.1	45.6	55.5	43.1	45.2
8	*Tepidimicrobium xylanilyticum* DSM 23310^{T} (GCF_900106765)	59.2	59.7	59.7	63.1	76.2	69.7	63.8	100	46.4	45.3	55.0	42.4	45.0
9	*Tissierella creatinini* BN11^{T} (GCF_005046945)	60.9	61.1	62.3	59.1	61.3	60.9	60.1	61.1	100	50.2	50.7	41.2	40.6
10	*Tissierella creatinophila* DSM 6911^{T} (GCF_001940565)	59.0	59.0	59.9	58.7	60.9	60.1	58.8	59.8	62.3	100	50.8	39.5	42.0
11	*Tissierella praeacuta* NCTC11158^{T} (GCF_900460335)	60.6	60.7	62.0	62.2	64.8	64.6	63.0	64.1	64.2	63.6	100	43.0	43.7
12	*Gottschalkia acidurici* 9a^{T} (GCF_000299355)	54.6	54.4	54.8	58.5	57.3	57.4	58.7	57.2	55.9	55.9	57.3	100	61.5
13	*Gottschalkia purinilytica* DSM 1384^{T} (GCF_001190785)	56.0	55.5	55.2	60.0	58.3	58.4	59.9	58.2	56.6	55.9	57.4	67.3	100
